# Radiomics of Tumor Heterogeneity in ^18^F-FDG-PET-CT for Predicting Response to Immune Checkpoint Inhibition in Therapy-Naïve Patients with Advanced Non-Small-Cell Lung Cancer

**DOI:** 10.3390/cancers15082297

**Published:** 2023-04-14

**Authors:** David Ventura, Philipp Schindler, Max Masthoff, Dennis Görlich, Matthias Dittmann, Walter Heindel, Michael Schäfers, Georg Lenz, Eva Wardelmann, Michael Mohr, Peter Kies, Annalen Bleckmann, Wolfgang Roll, Georg Evers

**Affiliations:** 1 Department of Nuclear Medicine, University Hospital Muenster, 48149 Muenster, Germany; 2 West German Cancer Center (WTZ), 48149 Muenster, Germany; 3 Clinic for Radiology, University and University Hospital Muenster, 48149 Muenster, Germany; 4 Institute of Biostatistics and Clinical Research, University of Muenster, 48149 Muenster, Germany; 5 Department of Medicine A-Hematology, Oncology, Hemostaseology and Pneumology, University Hospital Muenster, 48149 Muenster, Germany; 6 Gerhard-Domagk-Institute of Pathology, University Hospital Muenster, 48149 Muenster, Germany

**Keywords:** radiomics, FDG-PET-CT, NSCLC, PD-L1, PD-1, immune checkpoint inhibition, pembrolizumab, artificial intelligence, TPS

## Abstract

**Simple Summary:**

Biomarkers reliably predicting treatment response to immune checkpoint inhibition (CKI) therapy in advanced non-small cell lung cancer (NSCLC) are warranted. Baseline ^18^F-FDG-PET-CT (PET-CT) is an integral part of the diagnostic algorithm of NSCLC. However, there is poor evidence on the predictive and prognostic value of initial PET-CT imaging in these patients. The use of Radiomics has gained prominence in the last decade allowing for the extraction and artificial intelligence-based analysis of additional imaging parameters, so-called radiomic features (RFs). We aimed to find RFs predicting treatment response for CKI-based first-line therapy in advanced NSCLC patients, out of whole-body metabolic PET and morphological CT imaging. PET RFs might additionally be predictive and prognostic and could thus provide important information for future therapy monitoring and guidance.

**Abstract:**

We aimed to evaluate the predictive and prognostic value of baseline ^18^F-FDG-PET-CT (PET-CT) radiomic features (RFs) for immune checkpoint-inhibitor (CKI)-based first-line therapy in advanced non-small-cell lung cancer (NSCLC) patients. In this retrospective study 44 patients were included. Patients were treated with either CKI-monotherapy or combined CKI-based immunotherapy–chemotherapy as first-line treatment. Treatment response was assessed by the Response Evaluation Criteria in Solid Tumors (RECIST). After a median follow-up of 6.4 months patients were stratified into “responder” (*n* = 33) and “non-responder” (*n* = 11). RFs were extracted from baseline PET and CT data after segmenting PET-positive tumor volume of all lesions. A Radiomics-based model was developed based on a Radiomics signature consisting of reliable RFs that allow classification of response and overall progression using multivariate logistic regression. These RF were additionally tested for their prognostic value in all patients by applying a model-derived threshold. Two independent PET-based RFs differentiated well between responders and non-responders. For predicting response, the area under the curve (AUC) was 0.69 for “*PET-Skewness*” and 0.75 predicting overall progression for “*PET-Median*”. In terms of progression-free survival analysis, patients with a lower value of *PET-Skewness* (threshold < 0.2014; hazard ratio (HR) 0.17, 95% CI 0.06–0.46; *p* < 0.001) and higher value of *PET-Median* (threshold > 0.5233; HR 0.23, 95% CI 0.11–0.49; *p* < 0.001) had a significantly lower probability of disease progression or death. Our Radiomics-based model might be able to predict response in advanced NSCLC patients treated with CKI-based first-line therapy.

## 1. Introduction

Lung cancer is one of the most common cancers in men and women, with non-small cell lung cancer (NSCLC) accounting for up to 90% of primary lung tumors [1]. Unfortunately, more than 50% of patients are diagnosed at an advanced stage of disease (stage IV) and therefore have a limited survival rate [2]. In terms of therapy regimes, most patients have benefited only marginally from platinum-based therapy alone, which has been the standard of care for many decades [3].

Immune checkpoint inhibition (CKI) is a novel therapeutic option in early and advanced disease stages and has revolutionized anticancer treatment strategies [4]. Immune checkpoint signaling pathways allow tumor cells to evade immune surveillance resulting in tumor progression. Monoclonal antibodies targeting these signaling pathways boost host immunity against tumor cells by recruiting pre-existing tumor-specific cytotoxic T-cells [5]. Nowadays, monoclonal antibodies to the “programmed cell death protein 1” (PD-1) or to its ligand “programmed death-ligand 1” (PD-L1) either as monotherapy or in combination with chemotherapy are the standard of care for the treatment of advanced NSCLC patients [6].

Pivotal phase III trials have evaluated CKI in advanced NSCLC, both as monotherapy and in combination with chemotherapy as first-line treatment. Different clinical trials, which enrolled patients with advanced NSCLC and a high PD-L1 score (i.e., an immunohistochemical tumor proportion score (TPS) of at least 50%) compared CKI monotherapy with standard chemotherapy, showing a significant increase in progression-free (PFS) and overall survival (OS) in favor of the CKI-treated patient group [7,8,9]. For those patients with a TPS score < 50%, the addition of CKI to standard chemotherapy also demonstrated a significant survival benefit [10,11].

Despite advances in advanced NSCLC treatment, not all patients benefit equally from CKI-based therapy. Many patients suffering from early disease progression [11]. Against the background of high therapy costs and potential treatment-related side effects, better patient selection and identification of prognostic markers for treatment response and disease progression to CKI-based therapy is crucial. However, to date, it is still not possible to reliably predict treatment response in advanced NSCLC using biomarkers [12].

Today, [^18^F]-Fluorodeoxyglucose-(FDG)-positron emission tomography-computed tomography (further: PET-CT) is an integral part of the initial diagnostic work-up in NSCLC patients [13,14]. However, while PET-CT could be shown to have higher accuracy in tumor staging, most studies have failed to demonstrate prognostic value for various quantitative PET parameters [15]. The predictive value of imaging is limited as factors influencing therapeutic efficacy cannot be assessed using standard imaging parameters. Therefore, advanced imaging features which go beyond standard visual assessment are required to make further improvements. The extraction of high-throughput digital and quantitative imaging information and its conversion from encrypted imaging data to mineable numerical data allows its Radiomics analysis. Radiomics represents a groundbreaking new technique to analyze radiological data including the use of artificial intelligence that provides important insights into cancer phenotype and tumor heterogeneity [16,17,18]. In contrast to results on standard imaging assessment, several recently published studies found promising results on radiomic feature (RF)-based analysis in oncologic imaging for outcome prediction in several entities, including markers of tumor heterogeneity [16,19,20,21].

There is still limited evidence for Radiomics analysis using PET-CT imaging for predicting both the tumor expression of PD-L1 and the outcome in advanced NSCLC [22]. Hence, there is also limited evidence for the potential of Radiomics analysis to predict treatment response and outcome following first-line CKI-based treatment regimens in stage IV NSCLC patients. This is particularly true for studies not only including conventional CT imaging, but also PET-based RFs [16]. Because of the disseminated, highly heterogeneous disease manifestations, we included total tumor burden on PET-CT to identify significant RFs.

This study aimed to evaluate the benefit of a Radiomics-based model including quantitative morphological and metabolic information of PET-CT for predicting the response and survival of treatment-naïve patients with advanced NSCLC undergoing treatment with either CKI-monotherapy or CKI in combination with chemotherapy.

## 2. Materials and Methods

### 2.1. Study Design

This study was performed as a retrospective single-center observational trial in a tertiary care academic medical center. All patients received CKI with Pembrolizumab (Keytruda® Merck/MSD, Kenilworth, NJ, USA) as a monotherapy or in combination with chemotherapy according to pivotal study protocols. In patients treated with combined immunotherapy–chemotherapy, Pembrolizumab was combined with Cisplatin/Carboplatin and Pemetrexed for non-squamous carcinomas, whereas combination therapy with Carboplatin/Paclitaxel was administered for squamous cell carcinomas [23,24].

This study was approved by the local ethics committee (No. 2022-391-f-S, Ethics Commission of the Medical Association Westphalia-Lippe and the University Muenster). This study was performed in accordance with the ethical standards in the 1964 Declaration of Helsinki and its later amendments.

### 2.2. Patient Selection

The following inclusion criteria were applied to these patients to determine the study population: (a) histologically confirmed advanced NSCLC without driver alterations (only stage IV); (b) available TPS for PD-L1; (c) baseline PET-CT with available follow-up imaging data for response evaluation; (d) approval of the interdisciplinary lung tumor board for CKI-based therapy (assignment to TPS > 50%: CKI monotherapy, assignment to TPS < 50%: platinum-based immunotherapy chemotherapy); (e) no pretreatments; (f) age ≥ 18 years. 

### 2.3. Baseline ^18^F-FDG-PET-CT Imaging

Patients of the final cohort underwent a baseline PET-CT based on institutional standard protocols following current literature recommendations [13,25]. The imaging acquisition was performed using a Siemens Biograph mCT 128 System (Siemens Healthcare, Erlangen, Germany). All patients were imaged after a minimum of six hours of fasting with a blood glucose level < 6.7 mM. Images were acquired at 60 min after injection of 3 MBq/kg body weight of [^18^F]-FDG after appropriate standardized quality control. Whole-body images from skull base to proximal femur were acquired. An additional low-dose CT scan was performed in standard end-expiratory position for attenuation correction and anatomical correlation.

### 2.4. Response Assessment and Follow-Up

All patient and procedural data were retrospectively acquired from the electronic patient records as well as from the hospital’s image archiving and communications system. Electronic patient records have been reviewed for clinical data and therapy validations. Second follow-up imaging was performed to assess treatment response using a contrast-enhanced CT-scan. At this time, patients were classified by the RECIST (1.1) into “responders” (i.e., complete (CR)/partial response (PR) and stable disease (SD), mentioned as disease control rate (DCR)) and “non-responders” (i.e., progressive disease (PD)). CR was defined as a complete decrease in the primary lesion; PR as a decrease in the longest diameter by 20%; PD as an increase in the longest diameter by 30%; SD as neither a decrease nor an increase in the longest diameter as defined for PR or PD [26,27]. PFS was defined as time from starting CKI-based treatment until progression (PD) or death. Patients for whom follow-up information was not available after a certain time point were classified as “lost to follow-up”.

### 2.5. Image Segmentation and Feature Extraction

**Figure 1:**
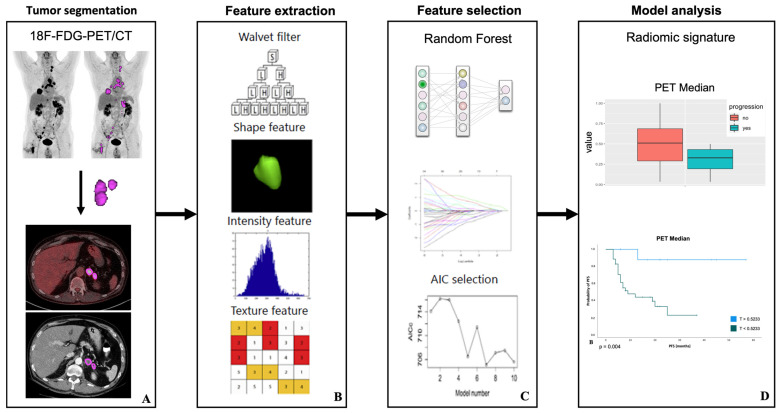
Radiomic features examination: PET positive volumes in baseline ^18^F-FDG-PET-CT were defined by application of a standardized threshold (PERCIST criteria) and manual adjustments, then transferred to low-dose CT dataset (**A**). RF extraction from a specific generated label map volume was done using an open source and multi-platform software package called 3D-Slicer, Version 4.11.2 (**B**). A Radiomics-based model was built based on a Radiomics signature consisting of reliable RFs that allow classification of second follow-up response using multivariate logistic regression (**C**). For predicting second follow-up response, the area under the receiver operating characteristic curve and the threshold of the Radiomics-based model was generated. These features were additionally tested for their prognostic value (PFS) with Kaplan–Meier and log-rank tests in all patients by applying a model-derived threshold (**D**). Modified to Yang et al. [30].

In this study the total tumor volume was initially defined based on a segmentation of the PET dataset. In agreement with previously published PERCIST criteria (1.1) a threshold was set to 1.5 × mean liver “standardized uptake value” (SUV) + 2 standard deviations to define FDG-positive tumor volume [28,29]. Two experienced nuclear medicine physicians, blinded for clinical data, each independently adjusted FDG-positive lesion volume manually and removed physiological uptakes, e. g., for liver, heart, and bladder. For image segmentation, the reader-specific label map volume, based on the PET-positive tumor volume, was then transferred to the CT images (Figure 1A). RFs from labelled PET and CT data were extracted twice, each by the same independent readers for inter-observer analysis. This included 36 first-order logic features and 48 gray level co-occurrence matrix (GLCM) features. These features are used to quantify tumor size (e.g., volume), shape (e.g., compactness and sphericity), and intensity (e.g., histogram statistics of mean, standard deviation and median) as well as texture matrices including the GCLM where the differences represent the heterogeneity of the tumor (Figure 1B). Image analysis and feature extraction was performed by using a freely available software package (3D slicer, version 4.11.2).

### 2.6. Feature Selection and Model Analysis

Feature selection and dimension reduction were necessary, as the number of RFs (*n* = 84) exceeded the number of patients (*n* = 44) [18,31]. The reproducibility of the extracted features between the two readers was assessed by calculating the concordance correlation coefficient (CCC) for each of the features as a measure of intra-class correlation. Features with a coefficient between 0.8 and 1 were classified as “excellent” and included in further analysis [32]. 

Using z-score standardization, all feature values were normalized to a range between 0 and 1, which improves comparability. The normalized dataset was randomly subdivided into a balanced training and test dataset (70/30 ratio). Further feature reduction was performed only on the training dataset using a Boruta machine learning algorithm. The Boruta algorithm applies a machine-learning-based random forest algorithm by making copies of all features that are called shadow features. Then, a random forest classifier is trained on this augmented dataset (original features plus shadow features) and the importance of each feature is evaluated. At each iteration, the Boruta algorithm checks whether a real feature has a higher importance. In doing so, it constantly removes features that are considered to be very unimportant. Finally, the Boruta algorithm stops when either all features are confirmed or discarded. On the other hand, Boruta finds all features that are either strongly or weakly relevant to the response variable (responder vs. non-responder) [33]. Subsequently, a correlation matrix was calculated since there is no relevant gain in information in closely correlated features (Figure 1C). 

Finally, to select features that allow either differentiation of responder and non-responder after the second follow-up or overall progression (see above) based on the training data set, a logistic regression analysis was performed on the test data set to fit and test the model. The discriminatory efficacy of the features was quantified by calculating the area under the curve (AUC) using receiver operating characteristic (ROC) by applying a model-derived threshold. Based on the ROC curve an optimal cut-off was defined using Youden’s index (Figure 1D) [34]. RF selection and dimension reduction was performed by using an open-source software package (R/R studio, version 4.0.5; R Foundation, Vienna, Austria).

### 2.7. Survival Analysis

After RF selection, significant parameters were tested for their prognostic value (PFS) with the Kaplan–Meier survival analysis. The log-rank test (Mantel–Cox) was used to assess between-group differences. Hazard ratios (HR) and associated 95% confidence intervals (95% CI) were calculated using a stratified Cox proportional hazards model. Higher TPS in advanced NSCLC patients treated with CKI-based first-line therapy and response to first-line therapy are known to be significantly associated with longer PFS [35,36,37]. These two clinical parameters were therefore additionally included in the survival analysis. 

### 2.8. Statistical Analysis

Clinical and demographic parameters were presented as total number, percentage, and range. *p*-values < 0.05 were set and considered to be statistically significant. Survival and statistical analysis were performed using the SPSS Statistics version 26 (SPSS Inc., Chicago, IL, USA). 

## 3. Results

### 3.1. Patients’ Characteristics

Between January 2017 and February 2022, a total of 44 patients with the initial diagnosis of stage IV NSCLC met all our above-mentioned inclusion criteria and were retrospectively analyzed. None of these patients had previously received therapy (i.e., thoracic radiotherapy, neoadjuvant or adjuvant therapy) for non-metastatic lung cancer. The median age of our patient cohort was 65 years (range: 35–82), and most patients were of male gender (70.5%). For the majority of patients (*n* = 30, 68.2%), continued (*n* = 10, 22.7%) or former (*n* = 20, 45.5%) smoking status could be documented at the time of initial NSCLC diagnosis. 

Of the 44 patients whose samples could be evaluated for PD-L1, 21 (47.7%) had a TPS of 50% or greater and all these patients underwent Pembrolizumab monotherapy. This compared with 13 patients (29.5%) and 10 patients (22.7%) with a TPS of 1–49% and <1%, respectively. In 35 patients (79.5%) pathologic work-up revealed non-squamous histology, whereas squamous carcinoma was found in 9 patients (20.5%). The demographic characteristics of the patients and the disease characteristics are summarized in Table 1.

### 3.2. Treatment

Following initial PET-CT imaging a Pembrolizumab-based therapy protocol was administered to all patients. According to current treatment guidelines 21 patients (47.7%) with TPS > 50% received Pembrolizumab monotherapy whereas 23 patients (53%) with a TPS < 50% received Pembrolizumab as combined immunotherapy chemotherapy. The median time from baseline PET-CT imaging to therapy start was 23 days (IQR: 22 days). All patients received at minimum four cycles of treatment with Pembrolizumab-based therapy. The median number of treatment cycles was 13 (range: 4–52).

### 3.3. Response and Clinical Outcome

**Figure 2:**
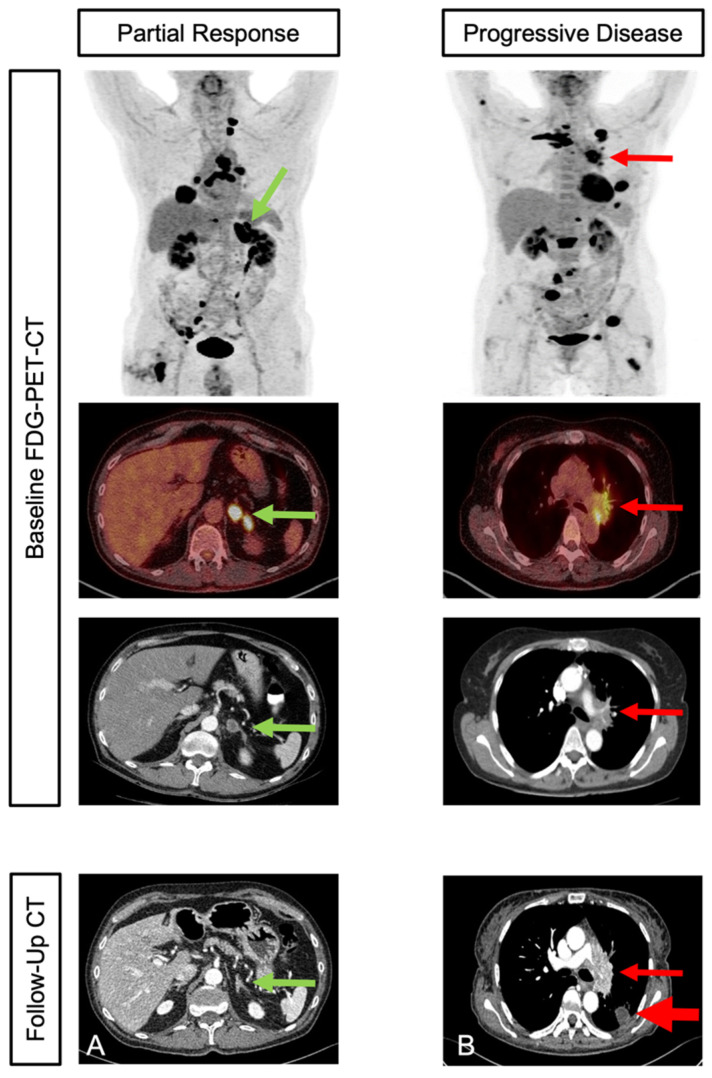
Initial staging and second follow-up in two patients with different response: Patient with adrenal (marked with green arrow), bone, and lymph node metastases in initial staging PET-CT. Partial response was shown in contrast-enhanced CT in second follow up (**A**). Patient with multiple intra-pulmonal (marked with red arrow) and bone metastases in initial staging PET-CT. Progressive disease was shown in contrast-enhanced CT in second follow up (**B**).

A second follow-up imaging with contrast-enhanced CT scans was performed to assess treatment response at a median of 6.4 months (95% CI, 6.2–6.7 months) (Figure 2). At this time, in accordance with the RECIST criteria, CR, PR, and SD were demonstrated in 1 patient (2.3%), 17 patients (38.6%), and 15 patients (34.1%), respectively. This contrasted with 10 patients (25%) who suffered from disease progression and were therefore classified as non-responders.

At the time of data cut-off 22 patients (50%) were still receiving assigned first-line treatment and 22 patients (50%) had received at least one subsequent therapy. With a median follow-up time of 18 months (range: 6–57), 22 patients suffered from disease progression (50.0%) and 10 patients (22.7%) died due to cancer-related circumstances. Hence, in our patient collective, based on 33 total events of progression or death, the median PFS was found to be 8 months (95% CI, 3–52).

### 3.4. Outcome Prediction

**Figure 3:**
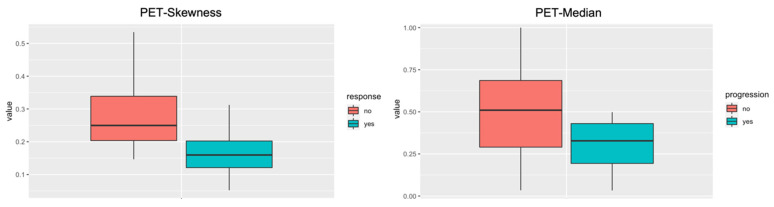
Radiomic features boxplot. The radiomic PET feature *PET-Skewness* differentiated well between non-responders (red) and responders (green) to immunotherapy after the second follow-up. The Radiomic PET feature *PET-Median* differentiated well between progression (green) and no-progression (red) overall.

Following data analysis, two radiomic PET features *(*“*PET-Skewness*” *and* “*PET-Median*”*)* revealed predictive value. *PET-Skewness* differentiated well between responder and non-responder at the second follow-up imaging (Figure 3). Hence, patients with a higher value and pronounced heterogeneity had a higher probability of disease progression at the second follow-up. *PET-Median* differentiated well between progression and no progression overall (Figure 3). Here, patients with a lower value had a higher probability of progressive disease following CKI-based first-line treatment.

For predicting response, the AUC-ROC of the Radiomics-based model was 0.69 for *PET-Skewness*. For predicting progression overall, the AUC-ROC of the Radiomics-based model was 0.75 for *PET-Median*.

### 3.5. Survival Analysis for PFS

**Figure 4:**
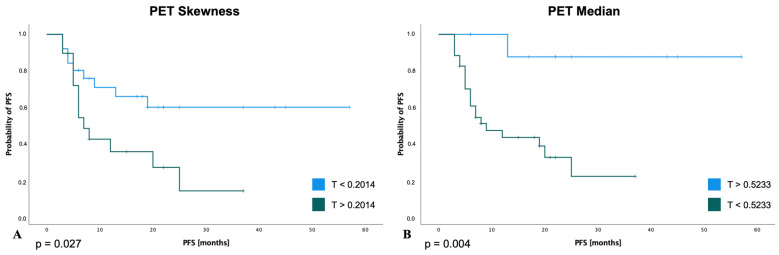

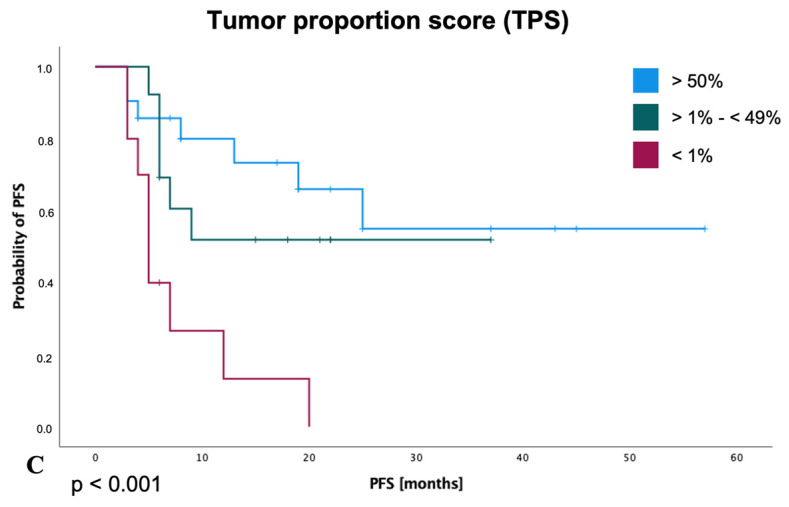
Progression-free survival for selected radiomic features and TPS score. Patients with a lower value of *PET-Skewness* with threshold (T): 0.2014 (**A**) and a higher value of *PET-Median* with threshold (T): 0.5233 (**B**) had a statistically significantly longer PFS. A higher TPS score (**C**) was also associated with a significantly longer PFS.

With respect to the entire patient population that was examined, within the final model of the Kaplan–Meier analysis and log-rank (Mantel–Cox) test, patients with a lower value of *PET-Skewness* (threshold 0.2014: 80.0% vs. 71.6%; *p* = 0.027) and higher value of *PET-Median* (threshold 0.5233: 93.3% vs. 45.7%; *p* = 0.004) had a significantly improved PFS at 6 months (Figure 4A,B). In addition, TPS (i.e., ≥50%, ≥1%–49%, and <1%) also showed a significant impact on PFS (*p* < 0.001) (Figure 4C). Using Cox regression analysis, patients with a lower value of *PET-Skewness* (threshold < 0.2014: HR 0.17, 95% CI 0.06–0.46; *p* < 0.001) and a higher value of *PET-median* (threshold > 0.5233: HR 0.23, 95% CI 0.11–0.49; *p* < 0.001) had a statistically significantly lower probability of disease progression or death. Similarly, TPS > 50% (HR 0.23, 95% CI 0.09–0.61; *p* = 0.003) and TPS 1–49% (HR 0.29, 95% CI 0.09–0.95; *p* = 0.041) were each associated with a lower likelihood of disease progression or death compared with a TPS < 1%.

**Figure 5:**
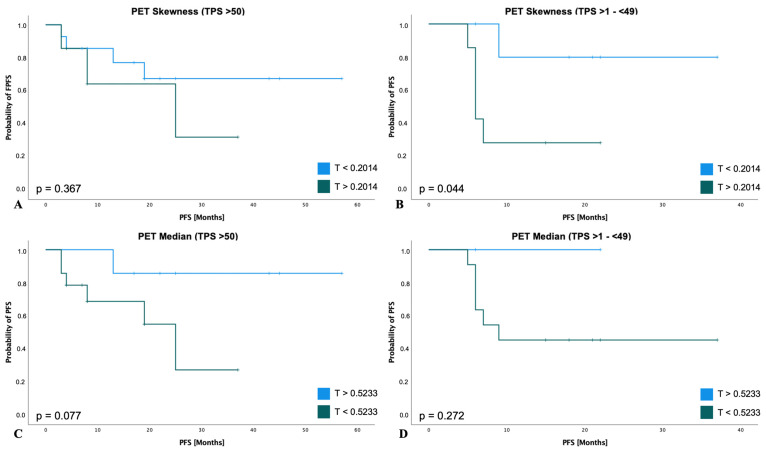
PFS subgroup analyses of RFs according to TPS. TPS > 50% subgroup (*n* = 21): For *PET-Skewness* (**A**) patients had a lower probability of disease progression or death with a value below a threshold of 0.2014 (HR: 0.57; 95% CI, 0.22 to 1.48; *p* = 0.247). For *PET-Median* (**B**) patients had a lower probability of disease progression or death with a value above a threshold of 0.5233 (HR: 0.26; 95% CI, 0.08 to 0.81; *p* = 0.021). TPS >1–(<49%) subgroup (*n* = 13): For *PET-Skewness* (**C**) patients had a lower probability of disease progression or death with a value below a threshold of 0.2014 (HR: 0.43; 95% CI, 0.13 to 1.40; *p* = 0.163). For *PET-Median* (**D**) patients had a lower probability of disease progression or death with a value above a threshold of 0.5233 (HR: 0.80; 95% CI, 0.17 to 3.76; *p* = 0.785).

The results of the subgroup analysis with respect to patients with a TPS score > 50% or a score of 1–49% are shown in Figure 5. Regarding the subgroup of patients with a TPS score > 50%, the parameter *PET-Skewness* also showed a trend in favor of patients with a value below the above-mentioned threshold (HR for disease progression or death, 0.57; 95% CI, 0.22 to 1.48; *p* = 0.247; Figure 5A). Similarly, in this group of patients, a lower probability of disease progression was shown for a *PET-Median* above the calculated threshold (HR for disease progression or death, 0.26; 95% CI, 0.08 to 0.81; *p* = 0.021; Figure 5C). When considering patients with a TPS score of 1–49%, the respective thresholds for *PET-Skewness* (HR 0.43; 95% CI, 0.13 to 1.40; *p* = 0.163; Figure 5B) and *PET-Median* (HR 0.80; 95% CI, 0.17 to 3.76; *p* = 0.785; Figure 5D) were also associated with a lower probability of disease progression.

## 4. Discussion

Despite novel therapeutic options, management of advanced NSCLC remains challenging in modern clinical oncology due to the limited response of therapy and disease progression in the majority of patients, resulting in high mortality rates [2]. However, over the past decades, treatment outcomes have significantly improved with the introduction of targeted therapies and CKI-based treatment regimens. In this context, numerous clinical trials have demonstrated a significant advantage of these new therapeutic regimens in terms of PFS and OS compared to conventional chemotherapies [38]. Although the introduction of CKI either as monotherapy or in combination with chemotherapy has improved the prognosis of NSCLC, still most patients die in the long-term course of their disease [39,40].

### 4.1. Radiomic Features as Potential Markers for Predicting Treatment Response and Survival

Immune checkpoint inhibitors interacting with PD1 and PD-L1 already showed improved OS for different tumor entities. Nevertheless, only 20–40% of patients benefit from this treatment option. Immunohistochemical assays are used to quantify PD-L1 in tumor cells to select appropriate patients. Hence, PD-L1 is the most studied, validated, and accepted biomarker according to current research. However, there are various challenges in clinical usage for this biomarker: (1) there is no unified standardized immunohistochemical assay; (2) the different assays show a high variance among each other, which leads to a higher variability; and (3) there is no prospective evidence between the different assays regarding treatment outcome [41].

For better patient management, it is crucial to identify other pretherapeutic markers that can predict the response to therapy and the outcome, thus enabling early treatment adjustments. Novel markers derived from initial patient imaging, such as PET-CT, could therefore provide a complementary option for guiding therapy and predicting treatment response and prognosis.

Analysis of our data suggests that imaging parameters derived from initial imaging before therapy initiation may have prognostic significance for evaluation of the response to therapy or disease progression. In this regard, our analysis identified significant differences for the overall population studied in terms of both treatment response at the second follow-up imaging (*PET-Skewness*) and PFS overall (*PET-Median*). 

Hence, an important clinical issue in the treatment of lung cancer patients, will be to select patients that will benefit from early intensification of therapy to improve response to the first-line treatment and to delay disease progression. In particular, imaging parameters may therefore be important for treatment guidance in patients with a TPS > 50% who may be treated with CKI alone or combined with chemotherapy according to the current guidelines [42].

Up to now, no prognostic marker has been identified to indicate which patients in this subgroup (TPS > 50%) will benefit from more intensive treatment with the addition of chemotherapy to CKI. Considering HR for disease progression, the results of our subgroup analysis suggest that the identified parameters may also have prognostic significance for this subgroup of patients. However, statistical significance could not be achieved here for all parameters and subgroups, which is probably due to the small number of patients included in our analysis. Our data must therefore be viewed with caution. Hence, analysis of larger patient collectives is essential for further investigation of our hypothesis.

### 4.2. PET-CT Derived Radiomic Features in Lung Cancer

PET parameters reflecting the whole tumor burden, such as the metabolic tumor volume or total lesion glycolysis, can be used as predictive parameters for lung cancer, as previously shown by [43,44,45,46,47]. However, these parameters often only indirectly represent the stage of the disease as, for example, a higher tumor volume is frequently associated with metastases. Especially when including patients with different stages of the disease in the study [19,20]. In contrast to studies including patients with different disease stages, PET-based tumor volume has not been identified to be a predictive factor for response to therapy in our study. Additional imaging-based parameters are needed to overcome these limitations, including information on tumor heterogeneity. Extracting additional information reflecting changes at the cellular level from metabolic and morphological imaging by Radiomics analysis might overcome these limitations. 

A systematic review by Morland et al. analyzed 107 different studies that addressed Radiomics in lung cancer based on PET-CT. However, the data on Radiomics-based approaches in advanced NSCLC patients treated with immunotherapy either as monotherapy or in combination with chemotherapy are still sparse [16]. In particular, investigations including both morphological and metabolic RFs equally, are rare [19,20,21]. Two of these studies with comparable populations included patients with several pretreatments before immunotherapy [19,20]. In these studies, some patients were diagnosed with stage IV at initial diagnosis, but some had progressed during or after previous therapies. However, it is known that progress during initial therapy is, a priori, a factor with a negative impact on survival [35,36,37]. Further, these two previous investigations did not subdivide datasets into a training and a test dataset, which is highly recommended when applying machine learning algorithms to validate RFs [48,49].

To avoid selection and performance biases, we therefore aimed to examine a treatment-naïve population that received CKI-based treatment as first-line therapy. The most valuable approach is presented in the study by Mu et al. (2020) with a retrospective analysis of a training and test dataset followed by an additional prospective validation of the RFs, resulting in a reliable assessment of their predictive power [21]. 

Although the primary advantage of whole-body PET imaging is the assessment of metabolic tumor properties in primary tumors and metastases, all three previous studies in contrast to our study only focused on the primary tumor for the assessment of RFs. However, in line with our results, all three previous publications identified radiomic metabolic features as significant for the prediction of PFS and OS. Moreover, these studies identified parameters reflecting tumor heterogeneity, albeit not identical to the predictive RFs identified in our study. Possible explanations are the above-mentioned differences in inclusion criteria, patient cohort, and segmentation for RFs of total tumor burden in baseline PET-CT. An advantage of PET RFs is their reproducibility and robustness to all degrading factors [17]. 

Regarding the morphological imaging component, no low-dose CT parameters were found to have significant predictive value concerning response or progression, which was comparable to the results from prior studies [19,20]. One bias could be the lower information output in low-dose CT compared to contrast-enhanced CT. In other studies, significant RFs were found in contrast-enhanced CT to predict therapy response to immunotherapy in advanced NSCLC [50,51]. Advances in CT techniques, such as photon counting CT, might further improve the assessment of tumor phenotype and should be combined with functional PET imaging in the future.

### 4.3. Limitations

This investigation is naturally limited due to its retrospective design and by its small patient cohort. Hence, a meaningful statistical analysis was not possible for the subgroups of patients that received either CKI monotherapy or combined immunotherapy–chemotherapy. Therefore, analyses of these subgroups in further investigations with more patients are warranted. Furthermore, due to the short follow-up period, no meaningful analysis of the impact of RFs on OS was performed in our investigation. Hence, future studies will therefore need to consider the impact of RFs on OS. 

To avoid bias through differences in background metabolic activity, instead of using a fixed threshold we used a reliable background-based quantification of tumor volume, as recommended in the PERCIST 1.1 criteria [28,29]. Open-source and deep learning programs for inter- and intra-observer and inter-software reliability are available and can minimize the bias of manually performed segmentation [33,52].

## 5. Conclusions

Our analysis suggests that PET-CT-based Radiomics may provide parameters with predictive value for response to first-line CKI-based treatment in patients with advanced NSCLC. Prospective studies are needed to translate a potential prognostic value of Radiomics analysis in this proof-of-concept study. Together with clinical and biological tumor-specific data, advanced image analysis could be a key element that might impact patient stratification and therapy guidance in advanced NSCLC patients.

## Figures and Tables

**Table 1:** Patients’ characteristics. Values are presented as median (interquartile range) or frequency (percentage).

Subjects	44	
Male	31	70.5%
Female	13	29.5%
**Age [Years]**	**65**	**(33–82)**
**Smoking status**		
Current	10	22.7%
Former	20	45.5%
Never	2	4.5%
Unknown	12	27.3%
**Eastern Cooperative Oncology Group (ECOG)**		
ECOG 0	2	4.5%
ECOG 1	32	72.7%
ECOG 2	10	22.7%
**Histology**		
Non-squamous carcinoma	35	79.5%
Squamous carcinoma	9	20.5%
**Tumor Proportion Score (TPS)-PD-L1**		
TPS > 50%	21	47.7%
TPS > 1%–(<49%)	13	29.5%
TPS < 1%	10	22.7%
**Response after Second Follow-Up (RECIST)**		
Complete Response (CR)	1	2.3%
Partial Response (PR)	17	38.6%
Stable Disease (SD)	15	34.1%
Progressive Disease (PD)	11	25.0%

## Data Availability

All data are included in the presented manuscript.
